# Crystal structure of the cubic double-perovskite Sr_2_Cr_0.84_Ni_0.09_Os_1.07_O_6_

**DOI:** 10.1107/S205698902201012X

**Published:** 2022-10-20

**Authors:** Jie Chen, Yoshihiro Tsujimoto, Alexei A. Belik, Kazunari Yamaura, Yoshitaka Matsushita

**Affiliations:** a National Institute for Materials Science, 1-1 Namiki, Tsukuba, Ibaraki 305-0044, Japan; University of Kentucky, USA

**Keywords:** crystal structure, osmate, oxide, high-pressure synthesis, cubic double perovskite

## Abstract

The crystal structure of the cubic double-perovskite Sr_2_Cr_0.84_Ni_0.09_Os_1.07_O_6_, grown at high pressure, was solved using intensity data measured at 113 K. The Os site was modelled with a partial Ni occupancy, and the Cr site was modelled with both Os and Ni partial occupancy. The refined structure shows that this cubic form is stable at 113 K.

## Abstract 2

The crystal structure of the cubic double-perovskite Sr_2_Cr_0.82_Ni_0.11_Os_1.07_O_6_ at 113 K is reported. The refined structure shows that this cubic form is stable at 113 K.

## 1. Chemical context

Recently, so called double-perovskites (DP) having *AB*′*B*′′O_6_ (*A* = divalent ions such as alkali earth or Pb, *B′*/*B*′′ = 3*d*/4*d*/*5d* transition metals) composition have attracted attention in the field of solid-state physics/chemistry due to their potential as materials for applications in, for example, spintronics, multiferroics, and/or magneto-caloric materials. In 1998, Sr_2_FeMoO_6_, which has the DP structure, was reported as having half-metallic behavior with a high Curie temperature (*T_C_* = 420 K) (Kobayashi *et al.*, 1998 ▸). After this discovery, many analogous DP compounds showing half-metallic and ferrimagnetic behavior have been reported (Table 1 ▸). The main contributors to the specific physical properties are the electronic states of the *B′* and *B*′′ elements. As an example, Sr_2_CrOsO_6_, which shows the highest *T_C_*, has its majority-spin orbital empty while the minority-spin orbital is fully occupied. Both Cr^3+^ (3*d* ^3^, *t_2g^3^_*) and Os^5+^ (5*d* ^3^, *t_2g^3^_*) activate primarily for this state (Mandal *et al.*, 2008 ▸). To enhance the property, we have introduced other transition metals into the *B′* and *B*′′ sites and examined for the exchange effects of such alternate transition metals at these sites. For this study, the samples were synthesized by high-pressure techniques; this was required to achieve the effective substitution.

## 2. Structural commentary

The crystal structure of Sr_2_Cr_0.84_Ni_0.09_Os_1.07_O_6_ has cubic symmetry of space group *Fm* 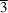 *m*, having one Sr, one Os, one Cr, and one O atom on crystallographically independent sites in the asymmetric unit. It corresponds to the fully Cr-containing end-member Sr_2_CrOsO_6_ and the low Ni-substituted Sr_2_Cr_0.75_Ni_0.25_OsO_6_ (Chen *et al.*, 2020 ▸), not the end-member of the Ni side of the composition, Sr_2_NiOsO_6_, which has tetra­gonal symmetry *I*4/*m* (Macquart *et al.*, 2005 ▸), or the high Ni-substituted Sr_2_Cr_0.50_Ni_0.50_OsO_6_ (HT: *I*4/*m* and LT: *C*2/*m*; Chen *et al.*, 2020 ▸).

**Figure 1:**
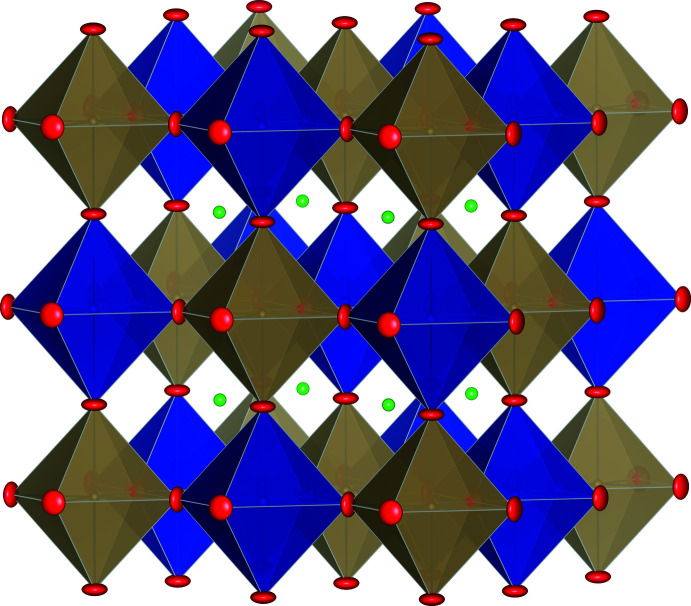
Displacement ellipsoid (probability 50%) and polyhedron view of the cubic double-perovskite Sr_2_Cr_0.84_Ni_0.09_Os_1.07_O_6_. Blue and light-brown polyhedra are CrO_6_ and OsO_6_, respectively. Red and green ellipsoids are oxygen and Sr, respectively. Figure drawn using *VESTA* (Momma & Izumi, 2011 ▸).

In the structure (Fig. 1 ▸), the transition metals located at both Cr (*B*′) and Os (*B*′′) sites show elemental disordering behavior: 96.1 (13)% Os + 3.8 (13)% Ni at the Os site and 85.5 (3)% Cr + 12.1 (3)% Os + 2.4 (3)% Ni at the Cr site. Both the Cr and Os sites form three-dimensional framework structures connected by corner sharing of the coordination octa­hedra, having Os—O = 1.926 (4) Å (coordination volume CV = 9.5405 Å^3^) and Cr—O = 1.987 (4) Å (CV = 10.4516 Å^3^) (Fig. 1 ▸). The Sr atoms, which are twelve coordinate, are located in the voids of the three-dimensional structure, Sr—O = 2.76739 (11) Å (CV: 49.9388 Å^3^). From this result, the cubic Sr_2_Cr_0.85_Ni_0.06_Os_1.08_O_6_ structure is shown to be stable down to at least 113K.

## 3. Synthesis and crystallization

A black-colored single crystal of Sr_2_Cr_0.84_Ni_0.09_Os_1.07_O_6_ was obtained as a by-product of the synthesis of the polycrystalline Sr_2_Cr_1-*x*_Ni_*x*_OsO_6_ (*x* = 0.5). The polycrystalline product was synthesized from powders of SrO (99.9%, Strem Chemicals, Inc., USA), CrO_2_ (Magtrieve, Sigma-Aldrich Co., USA), NiO (99.97%, High Purity Chemicals Co., Ltd., Japan), OsO_2_ [lab-made: Os powder (99.95%, Nanjing Dongrui Platinum Co., Ltd.) was heated at 673 under flowing O_2_ gas, the process was repeated three times]. The thoroughly mixed powders (SrO:CrO_2_:NiO: OsO_2_:KClO_4_ = 2:0.5:0.5:1:0.225 mol) were pressed into a pellet and sealed in a Pt capsule. All the processes were carried out in an Ar-filled glove box. A pressure of 6 GPa was continuously applied by a belt-type pressure apparatus (Kobe Steel, Ltd., Japan), the capsule was heated to 1873 K and held at that temperature for 1 h. The temperature was then quenched to room temperature, following which the pressure was gradually released.

## 4. Refinement

Crystal data, data collection and structure refinement details are summarized in Table 2 ▸. To ensure refinement stability, displacement parameters of disordered atoms on the same sites were constrained and the sums of occupancies were restrained (*SHELXL* commands EADP and SUMP, respectively.)

## Supplementary Material

Crystal structure: contains datablock(s) I, global. DOI: 10.1107/S205698902201012X/pk2669sup1.cif

Structure factors: contains datablock(s) I. DOI: 10.1107/S205698902201012X/pk2669Isup2.hkl

CCDC reference: 2213752

Additional supporting information:  crystallographic information; 3D view; checkCIF report

## Figures and Tables

**Table 1:** Typical half-metallic and ferrimagnetic double perovskites

Compound	Sr_2_FeMoO_6_	Sr_2_CrReO_6_	Sr_2_CrMoO_6_	Sr_2_FeReO_6_	Sr_2_CrWO_6_	Sr_2_CrOsO_6_
*T_C_* (K)	420	635	450	400	390	725 / 660
Reference	Kobayashi *et al.* (1998 ▸)	De Teresa *et al.* (2005 ▸) and Kato *et al.* (2002 ▸)	Moritomo *et al.* (2000 ▸)	Kobayashi *et al.* (1999 ▸)	Philipp *et al.* (2003 ▸)	Krockenberger *et al.* (2007 ▸) and Morrow *et al.* (2016 ▸)

**Table 2:** Experimental details

Crystal data	
Chemical formula	Cr_0.84_Ni_0.09_O_6_Os_1.07_Sr_2_
*M*_r_	524.37
Crystal system, space group	Cubic, *F* *m* 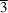 *m*
Temperature (K)	113
*a* (Å)	7.8269 (3)
*V* (Å^3^)	479.48 (6)
*Z*	4
Radiation type	Mo *K*α
μ (mm^−1^)	52.66
Crystal size (mm)	0.10 × 0.10 × 0.07

Data collection	
Diffractometer	Rigaku AFC11 Saturn724+ (4x4 bin mode)
Absorption correction	Multi-scan (*CrystalClear*; Rigaku, 2002 ▸)
*T*_min_, *T*_max_	0.056, 0.184
No. of measured, independent and observed [*I* > 2σ(*I*)] reflections	3159, 143, 143
*R*_int_	0.054
(sin θ/λ)_max_ (Å^−1^)	1.012

Refinement	
*R*[*F* ^2^ > 2σ(*F* ^2^)], *wR*(*F* ^2^), *S*	0.019, 0.047, 1.34
No. of reflections	143
No. of parameters	12
No. of restraints	1
Δρ_max_, Δρ_min_ (e Å^−3^)	2.87, −2.25

Computer programs: *CrystalClear* (Rigaku, 2002 ▸), *SHELXT2014/5* (Sheldrick, 2015*a* ▸), *SHELXL2018/1* (Sheldrick, 2015*b* ▸) and *VESTA* (Momma & Izumi, 2011 ▸).

## supplementary crystallographic information

### Crystal data

**Table:**

Cr_0.84_Ni_0.09_O_6_Os_1.07_Sr_2_	Mo *K*α radiation, λ = 0.71073 Å
*M**_r_* = 524.37	Cell parameters from 3313 reflections
Cubic, *F**m*3*m*	θ = 4.5–46.0°
*a* = 7.8269 (3) Å	µ = 52.66 mm^−^^1^
*V* = 479.48 (6) Å^3^	*T* = 113 K
*Z* = 4	Chunk, black
*F*(000) = 913	0.10 × 0.10 × 0.07 mm
*D*_x_ = 7.264 Mg m^−^^3^	

### Data collection

**Table:**

Rigaku AFC11 Saturn724+ (4x4 bin mode) diffractometer	143 independent reflections
Radiation source: Rigaku rotating anode	143 reflections with *I* > 2σ(*I*)
Confocal monochromator	*R*_int_ = 0.054
Detector resolution: 28.5714 pixels mm^-1^	θ_max_ = 46.0°, θ_min_ = 4.5°
dtprofit.ref scans	*h* = −15→11
Absorption correction: multi-scan (CrystalClear; Rigaku, 2002)	*k* = −15→11
*T*_min_ = 0.056, *T*_max_ = 0.184	*l* = −15→15
3159 measured reflections	

### Refinement

**Table:**

Refinement on *F*^2^	1 restraint
Least-squares matrix: full	*w* = 1/[σ^2^(*F*_o_^2^) + (0.0244*P*)^2^ + 3.1506*P*] where *P* = (*F*_o_^2^ + 2*F*_c_^2^)/3
*R*[*F*^2^ > 2σ(*F*^2^)] = 0.019	(Δ/σ)_max_ = 0.001
*wR*(*F*^2^) = 0.047	Δρ_max_ = 2.87 e Å^−^^3^
*S* = 1.34	Δρ_min_ = −2.25 e Å^−^^3^
143 reflections	Extinction correction: SHELXL-2018/1 (Sheldrick 2015b), Fc^*^=kFc[1+0.001xFc^2^λ^3^/sin(2θ)]^-1/4^
12 parameters	Extinction coefficient: 0.0047 (6)

### Special details

**Table:**

Geometry. All esds (except the esd in the dihedral angle between two l.s. planes) are estimated using the full covariance matrix. The cell esds are taken into account individually in the estimation of esds in distances, angles and torsion angles; correlations between esds in cell parameters are only used when they are defined by crystal symmetry. An approximate (isotropic) treatment of cell esds is used for estimating esds involving l.s. planes.

### Fractional atomic coordinates and isotropic or equivalent isotropic displacement parameters (Å^2^)

**Table:**

	*x*	*y*	*z*	*U*_iso_*/*U*_eq_	Occ. (<1)
Sr	0.250000	0.250000	0.250000	0.0075 (3)	
O	0.500000	0.500000	0.2461 (5)	0.0232 (9)	
Os	0.500000	0.500000	0.000000	0.00480 (13)	0.962 (13)
Ni'	0.500000	0.500000	0.000000	0.00480 (13)	0.037 (13)
Cr	0.500000	0.500000	0.500000	0.0065 (3)	0.838 (3)
Os'	0.500000	0.500000	0.500000	0.0065 (3)	0.112 (3)
Ni"	0.500000	0.500000	0.500000	0.0065 (3)	0.050 (3)

### Atomic displacement parameters (Å^2^)

**Table:**

	*U*^11^	*U*^22^	*U*^33^	*U*^12^	*U*^13^	*U*^23^
Sr	0.0075 (3)	0.0075 (3)	0.0075 (3)	0.000	0.000	0.000
O	0.0309 (14)	0.0309 (14)	0.0077 (13)	0.000	0.000	0.000
Os	0.00480 (13)	0.00480 (13)	0.00480 (13)	0.000	0.000	0.000
Ni'	0.00480 (13)	0.00480 (13)	0.00480 (13)	0.000	0.000	0.000
Cr	0.0065 (3)	0.0065 (3)	0.0065 (3)	0.000	0.000	0.000
Os'	0.0065 (3)	0.0065 (3)	0.0065 (3)	0.000	0.000	0.000
Ni"	0.0065 (3)	0.0065 (3)	0.0065 (3)	0.000	0.000	0.000

### Geometric parameters (Å, º)

**Table:**

Sr—O^i^	2.7674 (1)	Sr—O^ix^	2.7674 (1)
Sr—O^ii^	2.7674 (1)	Sr—O^x^	2.7674 (1)
Sr—O^iii^	2.7674 (1)	Sr—O^xi^	2.7674 (1)
Sr—O^iv^	2.7674 (1)	O—Ni'	1.926 (4)
Sr—O^v^	2.7674 (1)	O—Os	1.926 (4)
Sr—O	2.7674 (1)	O—Ni"	1.987 (4)
Sr—O^vi^	2.7674 (1)	O—Os'	1.987 (4)
Sr—O^vii^	2.7674 (1)	O—Cr	1.987 (4)
Sr—O^viii^	2.7674 (1)		
			
O^i^—Sr—O^ii^	58.98 (13)	O^ix^—Ni'—Sr^xv^	125.3
O^i^—Sr—O^iii^	119.996 (1)	O^xiv^—Ni'—Sr^xv^	54.7
O^ii^—Sr—O^iii^	178.75 (16)	O^xv^—Ni'—Sr^xv^	54.7
O^i^—Sr—O^iv^	58.98 (13)	Sr^xii^—Ni'—Sr^xv^	70.5
O^ii^—Sr—O^iv^	58.98 (13)	Sr^xvi^—Ni'—Sr^xv^	109.5
O^iii^—Sr—O^iv^	119.996 (1)	Sr—Ni'—Sr^xv^	180.0
O^i^—Sr—O^v^	119.996 (1)	Sr^x^—Ni'—Sr^xv^	109.5
O^ii^—Sr—O^v^	119.996 (1)	O—Ni'—Sr^xi^	54.7
O^iii^—Sr—O^v^	61.02 (13)	O^vii^—Ni'—Sr^xi^	54.7
O^iv^—Sr—O^v^	178.75 (16)	O^xiii^—Ni'—Sr^xi^	125.3
O^i^—Sr—O	178.75 (16)	O^ix^—Ni'—Sr^xi^	125.3
O^ii^—Sr—O	119.996 (1)	O^xiv^—Ni'—Sr^xi^	54.7
O^iii^—Sr—O	61.02 (13)	O^xv^—Ni'—Sr^xi^	125.3
O^iv^—Sr—O	119.996 (1)	Sr^xii^—Ni'—Sr^xi^	70.5
O^v^—Sr—O	61.02 (13)	Sr^xvi^—Ni'—Sr^xi^	109.5
O^i^—Sr—O^vi^	61.02 (13)	Sr—Ni'—Sr^xi^	70.5
O^ii^—Sr—O^vi^	90.007 (2)	Sr^x^—Ni'—Sr^xi^	109.5
O^iii^—Sr—O^vi^	90.007 (2)	Sr^xv^—Ni'—Sr^xi^	109.5
O^iv^—Sr—O^vi^	119.996 (1)	O^xvii^—Cr—O^iii^	180.0
O^v^—Sr—O^vi^	58.98 (13)	O^xvii^—Cr—O^xviii^	90.000 (1)
O—Sr—O^vi^	119.996 (1)	O^iii^—Cr—O^xviii^	90.0
O^i^—Sr—O^vii^	119.996 (1)	O^xvii^—Cr—O^v^	90.0
O^ii^—Sr—O^vii^	90.007 (2)	O^iii^—Cr—O^v^	90.000 (1)
O^iii^—Sr—O^vii^	90.007 (2)	O^xviii^—Cr—O^v^	180.0
O^iv^—Sr—O^vii^	61.02 (13)	O^xvii^—Cr—O^xix^	90.0
O^v^—Sr—O^vii^	119.996 (1)	O^iii^—Cr—O^xix^	90.0
O—Sr—O^vii^	58.98 (13)	O^xviii^—Cr—O^xix^	90.0
O^vi^—Sr—O^vii^	178.75 (16)	O^v^—Cr—O^xix^	90.0
O^i^—Sr—O^viii^	61.02 (13)	O^xvii^—Cr—O	90.0
O^ii^—Sr—O^viii^	119.996 (1)	O^iii^—Cr—O	90.0
O^iii^—Sr—O^viii^	58.98 (13)	O^xviii^—Cr—O	90.0
O^iv^—Sr—O^viii^	90.007 (2)	O^v^—Cr—O	90.0
O^v^—Sr—O^viii^	90.007 (2)	O^xix^—Cr—O	180.0
O—Sr—O^viii^	119.996 (1)	O^xvii^—Cr—Sr	125.3
O^vi^—Sr—O^viii^	61.02 (13)	O^iii^—Cr—Sr	54.7
O^vii^—Sr—O^viii^	119.996 (1)	O^xviii^—Cr—Sr	125.3
O^i^—Sr—O^ix^	119.996 (1)	O^v^—Cr—Sr	54.7
O^ii^—Sr—O^ix^	61.02 (13)	O^xix^—Cr—Sr	125.3
O^iii^—Sr—O^ix^	119.996 (1)	O—Cr—Sr	54.7
O^iv^—Sr—O^ix^	90.007 (2)	O^xvii^—Cr—Sr^xix^	54.7
O^v^—Sr—O^ix^	90.007 (2)	O^iii^—Cr—Sr^xix^	125.3
O—Sr—O^ix^	58.98 (13)	O^xviii^—Cr—Sr^xix^	54.7
O^vi^—Sr—O^ix^	119.996 (1)	O^v^—Cr—Sr^xix^	125.3
O^vii^—Sr—O^ix^	58.98 (13)	O^xix^—Cr—Sr^xix^	54.7
O^viii^—Sr—O^ix^	178.75 (16)	O—Cr—Sr^xix^	125.3
O^i^—Sr—O^x^	90.007 (2)	Sr—Cr—Sr^xix^	180.0
O^ii^—Sr—O^x^	61.02 (13)	O^xvii^—Cr—Sr^xi^	125.3
O^iii^—Sr—O^x^	119.996 (1)	O^iii^—Cr—Sr^xi^	54.7
O^iv^—Sr—O^x^	119.996 (1)	O^xviii^—Cr—Sr^xi^	54.7
O^v^—Sr—O^x^	58.98 (13)	O^v^—Cr—Sr^xi^	125.3
O—Sr—O^x^	90.007 (2)	O^xix^—Cr—Sr^xi^	125.264 (1)
O^vi^—Sr—O^x^	58.98 (13)	O—Cr—Sr^xi^	54.7
O^vii^—Sr—O^x^	119.996 (1)	Sr—Cr—Sr^xi^	70.5
O^viii^—Sr—O^x^	119.996 (1)	Sr^xix^—Cr—Sr^xi^	109.5
O^ix^—Sr—O^x^	61.02 (13)	O^xvii^—Cr—Sr^xx^	125.3
O^i^—Sr—O^xi^	90.007 (2)	O^iii^—Cr—Sr^xx^	54.7
O^ii^—Sr—O^xi^	119.996 (1)	O^xviii^—Cr—Sr^xx^	125.264 (1)
O^iii^—Sr—O^xi^	58.98 (13)	O^v^—Cr—Sr^xx^	54.7
O^iv^—Sr—O^xi^	61.02 (13)	O^xix^—Cr—Sr^xx^	54.7
O^v^—Sr—O^xi^	119.996 (1)	O—Cr—Sr^xx^	125.3
O—Sr—O^xi^	90.007 (2)	Sr—Cr—Sr^xx^	70.5
O^vi^—Sr—O^xi^	119.996 (1)	Sr^xix^—Cr—Sr^xx^	109.5
O^vii^—Sr—O^xi^	61.02 (13)	Sr^xi^—Cr—Sr^xx^	109.5
O^viii^—Sr—O^xi^	58.98 (13)	O^xvii^—Cr—Sr^xii^	54.7
O^ix^—Sr—O^xi^	119.996 (1)	O^iii^—Cr—Sr^xii^	125.3
O^x^—Sr—O^xi^	178.75 (16)	O^xviii^—Cr—Sr^xii^	54.7
Ni'—O—Os	0.0	O^v^—Cr—Sr^xii^	125.3
Ni'—O—Ni"	180.0	O^xix^—Cr—Sr^xii^	125.3
Os—O—Ni"	180.0	O—Cr—Sr^xii^	54.7
Ni'—O—Os'	180.0	Sr—Cr—Sr^xii^	109.5
Os—O—Os'	180.0	Sr^xix^—Cr—Sr^xii^	70.5
Ni"—O—Os'	0.0	Sr^xi^—Cr—Sr^xii^	70.5
Ni'—O—Cr	180.0	Sr^xx^—Cr—Sr^xii^	180.0
Os—O—Cr	180.0	O^xvii^—Cr—Sr^xxi^	125.3
Ni"—O—Cr	0.0	O^iii^—Cr—Sr^xxi^	54.7
Os'—O—Cr	0.0	O^xviii^—Cr—Sr^xxi^	54.7
Ni'—O—Sr^xii^	90.63 (8)	O^v^—Cr—Sr^xxi^	125.3
Os—O—Sr^xii^	90.63 (8)	O^xix^—Cr—Sr^xxi^	54.7
Ni"—O—Sr^xii^	89.37 (8)	O—Cr—Sr^xxi^	125.3
Os'—O—Sr^xii^	89.37 (8)	Sr—Cr—Sr^xxi^	109.5
Cr—O—Sr^xii^	89.37 (8)	Sr^xix^—Cr—Sr^xxi^	70.5
Ni'—O—Sr	90.63 (8)	Sr^xi^—Cr—Sr^xxi^	70.5
Os—O—Sr	90.63 (8)	Sr^xx^—Cr—Sr^xxi^	70.5
Ni"—O—Sr	89.37 (8)	Sr^xii^—Cr—Sr^xxi^	109.5
Os'—O—Sr	89.37 (8)	O^xvii^—Os'—O^iii^	180.0
Cr—O—Sr	89.37 (8)	O^xvii^—Os'—O^xviii^	90.000 (1)
Sr^xii^—O—Sr	178.75 (16)	O^iii^—Os'—O^xviii^	90.0
Ni'—O—Sr^x^	90.63 (8)	O^xvii^—Os'—O^v^	90.0
Os—O—Sr^x^	90.63 (8)	O^iii^—Os'—O^v^	90.000 (1)
Ni"—O—Sr^x^	89.37 (8)	O^xviii^—Os'—O^v^	180.0
Os'—O—Sr^x^	89.37 (8)	O^xvii^—Os'—O^xix^	90.0
Cr—O—Sr^x^	89.37 (8)	O^iii^—Os'—O^xix^	90.0
Sr^xii^—O—Sr^x^	89.993 (2)	O^xviii^—Os'—O^xix^	90.0
Sr—O—Sr^x^	89.993 (2)	O^v^—Os'—O^xix^	90.0
Ni'—O—Sr^xi^	90.63 (8)	O^xvii^—Os'—O	90.0
Os—O—Sr^xi^	90.63 (8)	O^iii^—Os'—O	90.0
Ni"—O—Sr^xi^	89.37 (8)	O^xviii^—Os'—O	90.0
Os'—O—Sr^xi^	89.37 (8)	O^v^—Os'—O	90.0
Cr—O—Sr^xi^	89.37 (8)	O^xix^—Os'—O	180.0
Sr^xii^—O—Sr^xi^	89.993 (2)	O^xvii^—Os'—Sr	125.3
Sr—O—Sr^xi^	89.993 (2)	O^iii^—Os'—Sr	54.7
Sr^x^—O—Sr^xi^	178.75 (16)	O^xviii^—Os'—Sr	125.3
O—Os—O^vii^	90.0	O^v^—Os'—Sr	54.7
O—Os—O^xiii^	90.0	O^xix^—Os'—Sr	125.3
O^vii^—Os—O^xiii^	180.0	O—Os'—Sr	54.7
O—Os—O^ix^	90.0	O^xvii^—Os'—Sr^xix^	54.7
O^vii^—Os—O^ix^	90.0	O^iii^—Os'—Sr^xix^	125.3
O^xiii^—Os—O^ix^	90.0	O^xviii^—Os'—Sr^xix^	54.7
O—Os—O^xiv^	90.0	O^v^—Os'—Sr^xix^	125.3
O^vii^—Os—O^xiv^	90.0	O^xix^—Os'—Sr^xix^	54.7
O^xiii^—Os—O^xiv^	90.0	O—Os'—Sr^xix^	125.3
O^ix^—Os—O^xiv^	180.0	Sr—Os'—Sr^xix^	180.0
O—Os—O^xv^	180.0	O^xvii^—Os'—Sr^xi^	125.3
O^vii^—Os—O^xv^	90.0	O^iii^—Os'—Sr^xi^	54.7
O^xiii^—Os—O^xv^	90.0	O^xviii^—Os'—Sr^xi^	54.7
O^ix^—Os—O^xv^	90.0	O^v^—Os'—Sr^xi^	125.3
O^xiv^—Os—O^xv^	90.0	O^xix^—Os'—Sr^xi^	125.264 (1)
O—Os—Sr^xii^	54.7	O—Os'—Sr^xi^	54.7
O^vii^—Os—Sr^xii^	125.3	Sr—Os'—Sr^xi^	70.5
O^xiii^—Os—Sr^xii^	54.7	Sr^xix^—Os'—Sr^xi^	109.5
O^ix^—Os—Sr^xii^	125.3	O^xvii^—Os'—Sr^xx^	125.3
O^xiv^—Os—Sr^xii^	54.7	O^iii^—Os'—Sr^xx^	54.7
O^xv^—Os—Sr^xii^	125.3	O^xviii^—Os'—Sr^xx^	125.264 (1)
O—Os—Sr^xvi^	125.3	O^v^—Os'—Sr^xx^	54.7
O^vii^—Os—Sr^xvi^	54.7	O^xix^—Os'—Sr^xx^	54.7
O^xiii^—Os—Sr^xvi^	125.3	O—Os'—Sr^xx^	125.3
O^ix^—Os—Sr^xvi^	54.7	Sr—Os'—Sr^xx^	70.5
O^xiv^—Os—Sr^xvi^	125.3	Sr^xix^—Os'—Sr^xx^	109.5
O^xv^—Os—Sr^xvi^	54.7	Sr^xi^—Os'—Sr^xx^	109.5
Sr^xii^—Os—Sr^xvi^	180.0	O^xvii^—Os'—Sr^xii^	54.7
O—Os—Sr	54.7	O^iii^—Os'—Sr^xii^	125.3
O^vii^—Os—Sr	54.7	O^xviii^—Os'—Sr^xii^	54.7
O^xiii^—Os—Sr	125.3	O^v^—Os'—Sr^xii^	125.3
O^ix^—Os—Sr	54.7	O^xix^—Os'—Sr^xii^	125.3
O^xiv^—Os—Sr	125.3	O—Os'—Sr^xii^	54.7
O^xv^—Os—Sr	125.3	Sr—Os'—Sr^xii^	109.5
Sr^xii^—Os—Sr	109.5	Sr^xix^—Os'—Sr^xii^	70.5
Sr^xvi^—Os—Sr	70.5	Sr^xi^—Os'—Sr^xii^	70.5
O—Os—Sr^x^	54.7	Sr^xx^—Os'—Sr^xii^	180.0
O^vii^—Os—Sr^x^	125.3	O^xvii^—Os'—Sr^xxi^	125.3
O^xiii^—Os—Sr^x^	54.7	O^iii^—Os'—Sr^xxi^	54.7
O^ix^—Os—Sr^x^	54.7	O^xviii^—Os'—Sr^xxi^	54.7
O^xiv^—Os—Sr^x^	125.3	O^v^—Os'—Sr^xxi^	125.3
O^xv^—Os—Sr^x^	125.3	O^xix^—Os'—Sr^xxi^	54.7
Sr^xii^—Os—Sr^x^	70.5	O—Os'—Sr^xxi^	125.3
Sr^xvi^—Os—Sr^x^	109.5	Sr—Os'—Sr^xxi^	109.5
Sr—Os—Sr^x^	70.5	Sr^xix^—Os'—Sr^xxi^	70.5
O—Os—Sr^xv^	125.3	Sr^xi^—Os'—Sr^xxi^	70.5
O^vii^—Os—Sr^xv^	125.3	Sr^xx^—Os'—Sr^xxi^	70.5
O^xiii^—Os—Sr^xv^	54.7	Sr^xii^—Os'—Sr^xxi^	109.5
O^ix^—Os—Sr^xv^	125.3	O^xvii^—Ni"—O^iii^	180.0
O^xiv^—Os—Sr^xv^	54.7	O^xvii^—Ni"—O^xviii^	90.000 (1)
O^xv^—Os—Sr^xv^	54.7	O^iii^—Ni"—O^xviii^	90.0
Sr^xii^—Os—Sr^xv^	70.5	O^xvii^—Ni"—O^v^	90.0
Sr^xvi^—Os—Sr^xv^	109.5	O^iii^—Ni"—O^v^	90.000 (1)
Sr—Os—Sr^xv^	180.0	O^xviii^—Ni"—O^v^	180.0
Sr^x^—Os—Sr^xv^	109.5	O^xvii^—Ni"—O^xix^	90.0
O—Os—Sr^xi^	54.7	O^iii^—Ni"—O^xix^	90.0
O^vii^—Os—Sr^xi^	54.7	O^xviii^—Ni"—O^xix^	90.0
O^xiii^—Os—Sr^xi^	125.3	O^v^—Ni"—O^xix^	90.0
O^ix^—Os—Sr^xi^	125.3	O^xvii^—Ni"—O	90.0
O^xiv^—Os—Sr^xi^	54.7	O^iii^—Ni"—O	90.0
O^xv^—Os—Sr^xi^	125.3	O^xviii^—Ni"—O	90.0
Sr^xii^—Os—Sr^xi^	70.5	O^v^—Ni"—O	90.0
Sr^xvi^—Os—Sr^xi^	109.5	O^xix^—Ni"—O	180.0
Sr—Os—Sr^xi^	70.5	O^xvii^—Ni"—Sr	125.3
Sr^x^—Os—Sr^xi^	109.5	O^iii^—Ni"—Sr	54.7
Sr^xv^—Os—Sr^xi^	109.5	O^xviii^—Ni"—Sr	125.3
O—Ni'—O^vii^	90.0	O^v^—Ni"—Sr	54.7
O—Ni'—O^xiii^	90.0	O^xix^—Ni"—Sr	125.3
O^vii^—Ni'—O^xiii^	180.0	O—Ni"—Sr	54.7
O—Ni'—O^ix^	90.0	O^xvii^—Ni"—Sr^xix^	54.7
O^vii^—Ni'—O^ix^	90.0	O^iii^—Ni"—Sr^xix^	125.3
O^xiii^—Ni'—O^ix^	90.0	O^xviii^—Ni"—Sr^xix^	54.7
O—Ni'—O^xiv^	90.0	O^v^—Ni"—Sr^xix^	125.3
O^vii^—Ni'—O^xiv^	90.0	O^xix^—Ni"—Sr^xix^	54.7
O^xiii^—Ni'—O^xiv^	90.0	O—Ni"—Sr^xix^	125.3
O^ix^—Ni'—O^xiv^	180.0	Sr—Ni"—Sr^xix^	180.0
O—Ni'—O^xv^	180.0	O^xvii^—Ni"—Sr^xi^	125.3
O^vii^—Ni'—O^xv^	90.0	O^iii^—Ni"—Sr^xi^	54.7
O^xiii^—Ni'—O^xv^	90.0	O^xviii^—Ni"—Sr^xi^	54.7
O^ix^—Ni'—O^xv^	90.0	O^v^—Ni"—Sr^xi^	125.3
O^xiv^—Ni'—O^xv^	90.0	O^xix^—Ni"—Sr^xi^	125.264 (1)
O—Ni'—Sr^xii^	54.7	O—Ni"—Sr^xi^	54.7
O^vii^—Ni'—Sr^xii^	125.3	Sr—Ni"—Sr^xi^	70.5
O^xiii^—Ni'—Sr^xii^	54.7	Sr^xix^—Ni"—Sr^xi^	109.5
O^ix^—Ni'—Sr^xii^	125.3	O^xvii^—Ni"—Sr^xx^	125.3
O^xiv^—Ni'—Sr^xii^	54.7	O^iii^—Ni"—Sr^xx^	54.7
O^xv^—Ni'—Sr^xii^	125.3	O^xviii^—Ni"—Sr^xx^	125.264 (1)
O—Ni'—Sr^xvi^	125.3	O^v^—Ni"—Sr^xx^	54.7
O^vii^—Ni'—Sr^xvi^	54.7	O^xix^—Ni"—Sr^xx^	54.7
O^xiii^—Ni'—Sr^xvi^	125.3	O—Ni"—Sr^xx^	125.3
O^ix^—Ni'—Sr^xvi^	54.7	Sr—Ni"—Sr^xx^	70.5
O^xiv^—Ni'—Sr^xvi^	125.3	Sr^xix^—Ni"—Sr^xx^	109.5
O^xv^—Ni'—Sr^xvi^	54.7	Sr^xi^—Ni"—Sr^xx^	109.5
Sr^xii^—Ni'—Sr^xvi^	180.0	O^xvii^—Ni"—Sr^xii^	54.7
O—Ni'—Sr	54.7	O^iii^—Ni"—Sr^xii^	125.3
O^vii^—Ni'—Sr	54.7	O^xviii^—Ni"—Sr^xii^	54.7
O^xiii^—Ni'—Sr	125.3	O^v^—Ni"—Sr^xii^	125.3
O^ix^—Ni'—Sr	54.7	O^xix^—Ni"—Sr^xii^	125.3
O^xiv^—Ni'—Sr	125.3	O—Ni"—Sr^xii^	54.7
O^xv^—Ni'—Sr	125.3	Sr—Ni"—Sr^xii^	109.5
Sr^xii^—Ni'—Sr	109.5	Sr^xix^—Ni"—Sr^xii^	70.5
Sr^xvi^—Ni'—Sr	70.5	Sr^xi^—Ni"—Sr^xii^	70.5
O—Ni'—Sr^x^	54.7	Sr^xx^—Ni"—Sr^xii^	180.0
O^vii^—Ni'—Sr^x^	125.3	O^xvii^—Ni"—Sr^xxi^	125.3
O^xiii^—Ni'—Sr^x^	54.7	O^iii^—Ni"—Sr^xxi^	54.7
O^ix^—Ni'—Sr^x^	54.7	O^xviii^—Ni"—Sr^xxi^	54.7
O^xiv^—Ni'—Sr^x^	125.3	O^v^—Ni"—Sr^xxi^	125.3
O^xv^—Ni'—Sr^x^	125.3	O^xix^—Ni"—Sr^xxi^	54.7
Sr^xii^—Ni'—Sr^x^	70.5	O—Ni"—Sr^xxi^	125.3
Sr^xvi^—Ni'—Sr^x^	109.5	Sr—Ni"—Sr^xxi^	109.5
Sr—Ni'—Sr^x^	70.5	Sr^xix^—Ni"—Sr^xxi^	70.5
O—Ni'—Sr^xv^	125.3	Sr^xi^—Ni"—Sr^xxi^	70.5
O^vii^—Ni'—Sr^xv^	125.3	Sr^xx^—Ni"—Sr^xxi^	70.5
O^xiii^—Ni'—Sr^xv^	54.7	Sr^xii^—Ni"—Sr^xxi^	109.5

Symmetry codes: (i) *x*−1/2, *y*−1/2, *z*; (ii) *y*−1/2, *z*, *x*−1/2; (iii) *y*, *z*, *x*; (iv) *z*, *x*−1/2, *y*−1/2; (v) *z*, *x*, *y*; (vi) −*y*+1/2, −*z*+1/2, −*x*+1; (vii) −*y*+1, −*z*+1/2, −*x*+1/2; (viii) −*z*+1/2, −*x*+1/2, −*y*+1; (ix) −*z*+1/2, −*x*+1, −*y*+1/2; (x) −*x*+1/2, −*y*+1, −*z*+1/2; (xi) −*x*+1, −*y*+1/2, −*z*+1/2; (xii) *x*+1/2, *y*+1/2, *z*; (xiii) *y*, *z*+1/2, *x*−1/2; (xiv) *z*+1/2, *x*, *y*−1/2; (xv) −*x*+1, −*y*+1, −*z*; (xvi) −*x*+1/2, −*y*+1/2, −*z*; (xvii) −*y*+1, −*z*+1, −*x*+1; (xviii) −*z*+1, −*x*+1, −*y*+1; (xix) −*x*+1, −*y*+1, −*z*+1; (xx) −*x*+1/2, −*y*+1/2, −*z*+1; (xxi) *x*+1/2, *y*, *z*+1/2.
